# University engagement of dental students related to educational environment: A transnational study

**DOI:** 10.1371/journal.pone.0259524

**Published:** 2021-11-04

**Authors:** Cristina Dupim Presoto, Danielle Wajngarten, Patrícia Aleixo dos Santos Domingos, Ana Carolina Botta, Juliana Alvares Duarte Bonini Campos, Júlia Margato Pazos, Patrícia Petromilli Nordi Sasso Garcia

**Affiliations:** 1 Department of Social Dentistry, São Paulo State University (Unesp), School of Dentistry, Araraquara, São Paulo, Brazil; 2 Department of Dentistry, University of Araraquara (UNIARA), School of Dentistry, Araraquara, São Paulo, Brazil; 3 Department of General Dentistry, Stony Brook School of Dental Medicine, New York, New York, United States of America; 4 Department of Food and Nutrition, São Paulo State University (Unesp), School of Pharmaceutical Sciences, Araraquara, São Paulo, Brazil; Centre Hospitalier Regional Universitaire de Tours, FRANCE

## Abstract

**Objectives:**

To investigate the validity and reliability of the University Student Engagement Inventory (USEI) in its complete and reduced versions with Brazilian and USA students, and to evaluate the influence of gender and academic level on students’ university engagement in both countries.

**Methods:**

A cross-sectional observational study with a non-probability sample was conducted. The sample comprised dental students of both genders, 154 from a university in New York, USA (response rate 91.1%) and 459 from two universities in Brazil (response rate 79.1%). University engagement was measured using the USEI. The samples were characterized by gender and academic level.

**Results:**

The refined reduced version of the USEI presented adequate fit to the samples from both countries. Gender was associated with the behavioral engagement factor of the USEI in Brazilian students. In the USA sample, gender was associated with the behavioral and cognitive engagement factors. There was a significant effect of academic level on behavioral and emotional engagement for the Brazilian and USA samples, respectively.

**Conclusion:**

The refined reduced USEI presented adequate psychometric qualities for the study samples.

## Introduction

The engagement of university students with issues related to their educational environment is fundamental for the improvement of their learning experience and overall well-being [1] and may have positive effects on their future career. According to Te Brake et al. [2] and Jugale et al. [3], engagement in school and later at work has protective effects for emotional health, since individuals involved with their tasks have lower levels of emotional exhaustion and depersonalization and higher levels of personal achievement.

Two psychometric scales have been used to measure the university engagement construct, the Utrecht Work Engagement Scale for Students (UWES-SS) [4] and University Student Engagement Inventory (USEI) [1]. The USEI consists of 32 items distributed in a three-factor structure covering behavioral, emotional, and cognitive engagement [1]. Behavioral engagement refers to student participation in both classroom and extracurricular activities; emotional engagement covers students’ feelings of agreement or disagreement with teachers’ instructions, and cognitive engagement refers to the commitment and vigor expended by students to understand complex concepts and put them into practice [1,5]. The 32-item USEI, originally proposed by Maroco et al. [1], did not show a good adjustment when validated with Portuguese university students in the areas of social, biological, and exact sciences, and engineering. Therefore, the authors refined the instrument and obtained a reduced scale of tri-factorial structure containing 15 items. In Brazil, this instrument was validated with pharmacy-biochemistry students [5].

To date, no studies evaluating university engagement among dental students have been conducted using the USEI. On the other hand, the engagement of graduated dental professionals with their work activities has been evaluated, evidencing good levels of involvement [2,3,6–8]. According to Gorter et al. [7], greater engagement of dental professionals with their work resulted in a better relationship with the patient, greater satisfaction with the results of the treatment performed, and greater enjoyment and satisfaction with their performance.

Considering the importance of university engagement in maintaining mental health and preventing high levels of stress among students, this study aimed to determine the validity and reliability of the USEI, in its original and reduced versions, with USA and Brazilian students, and to evaluate the influence of gender and academic level on university engagement.

## Materials and methods

### Sample and study design

This was an observational cross-sectional study using non-probability sampling. All undergraduate dental students at Stony Brook School of Dental Medicine, Stony Brook University (SBU) in New York, USA (n = 169) and two Brazilian dental schools, UNESP School of Dentistry in Araraquara (n = 375) and University of Araraquara (UNIARA) School of Dentistry (n = 205), were invited to participate in the study. Only the students who agreed to participate and signed the informed consent form were included in the study (UNESP: n = 293, response rate = 78.1%; UNIARA: n = 166, response rate = 81.0%; and SBU: n = 154, response rate = 91.1%).

The minimum sample size was estimated following the recommendations by Kim [9], that suggest using the test power. Thus, considering a 5% significance level, 80% power, and 461 degrees of freedom (df), the minimum sample size estimated for this study was 82 participants in each group. As the present study was conducted in an educational setting, all students were invited to participate, thus obtaining a larger sample size than the calculated minimum.

This study was approved by the Research Ethics Committee of the School of Dentistry of São Paulo State University (UNESP), Araraquara Campus (CAAE Registry No. 4753816.9.0000.5416) and by the Stony Brook University Institutional Review Board (IRB) (CORIHS# 2016-3444-F).

### Study variables and measuring instrument

The student samples were characterized by gender and academic level. Students’ university engagement was evaluated using the USEI, originally developed in Portuguese and validated for students in Portugal [1]. The USEI is composed of 32 items assessed on a 5-point scale ranging from 1 (never) to 5 (always). The items are distributed as follows: behavioral engagement (items 1–11), emotional engagement (items 12–21), and cognitive engagement (items 22–32). The instrument’s reduced version is composed of 15 of the original 32 items, assessed in the same way as in the complete instrument. In the short version, the behavioral engagement factor contains items 1, 3, 4, 5, and 6, the emotional engagement factor items 14, 15, 16, 17, and 19, and the cognitive engagement factor items 22, 25, 26, 28, and 32.

The 32-item version of the USEI was administered to students who agreed to participate in the study. The questionnaires were completed in the classroom, in the presence of the researcher.

The average filling time of the questionnaire was about 15 minutes. Questionnaires with any missing data were not included in this study.

### Statistical analysis

#### Psychometric sensitivity

The measurements of summary and shape was used to evaluate Psychometric sensitivity. It was considered appropriate when skewness was <3 and kurtosis was < 7, in terms of their absolute values.

#### Construct validity

Construct validity of the USEI was estimated by means of factorial, convergent, and discriminant validities. Factorial validity was evaluated through confirmatory factor analysis using a polychoric correlation matrix with weighted least squares mean and variance adjusted estimation. The chi-square by degrees of freedom ratio (χ^2^/df), comparative fit index (CFI), Tucker Lewis index (TLI), root mean square error of approximation (RMSEA), and weighted root mean square residual value (WRMR) were used as goodness-of-fit indices [10,11]. Global fit was considered adequate when χ^2^/df≤2.0, CFI and TLI≥0.90, and RMSEA≤0.10, and local fit was considered adequate when lambda values (λ) ≥0.40 [10,11]. WRMR values of <0.08 were also considered as indication of good fit [11]. Items with λ <0.40 were removed. If the model did not show an adequate fit to the data, the modification indices (estimated using the Lagrange Multiplier [LM] method) with values above 11 were inspected to check for any correlations between errors of items [12,13].

Convergent validity was assessed using average variance extracted (AVE) [14], and was considered adequate if AVE_j_≥0.50 [11,15]. Discriminant validity was evaluated through an analysis of the correlations between the factors (i,j). The discriminant validity was considered adequate when AVE values of the correlated factors were above or equal to the squared correlation between factors (AVE_i_ and AVE_j_≥rij^2^).

#### Reliability

Reliability was estimated using composite reliability (CR) and Cronbach’s alpha coefficient (α). The latter was calculated using a polychoric correlation matrix created with the R^®^ software (Core Team, 2016). Reliability was considered adequate when α and CR values were ≥0.70 [13,14].

#### Structural model

In order to verify the contribution of gender and academic level to dental students’ university engagement, a causal model was elaborated using structural equation modeling. The model was assessed in two steps using the MPLUS 7.2 software program (Muthén & Muthén, Los Angeles, USA). In the first step, the model’s quality of fit was evaluated using χ^2^/df, CFI, TLI, and RMSEA values. The model fit was considered adequate using the same parameters described for the instrument’s validation process.^11^ In the second stage, the Z-test was used to assess the contribution and significance of trajectories (β) using a 5% significance level.

All analyses were performed using the Statistical Package for the Social Sciences (SPSS) software, version 22.0, the MPlus software, version 7.2 (Muthén & Muthén, Los Angeles, USA), and the R^®^ software (Core Team, 2016).

## Results

Table 1 shows the demographic characteristics of the study samples.

**Table 1:** Demographic characteristics of the study samples.

Variable	Country		
	Brazil n = 459	United States n = 154	Total N = 613
**Gender**			
**Female**	338 (73.6%)	84 (54.6%)	422 (68.8%)
**Male**	121 (26.4%)	70 (45.4%)	191 (31.2%)
**Academic Level**			
**1**^**st**^ **Year**	109 (23.7%)	41 (26.6%)	150 (24.5%)
**2**^**nd**^ **Year**	104 (22.7%)	43 (27.9%)	147 (24.0%)
**3**^**rd**^ **Year**	106 (23.1%)	37 (24.1%)	143 (23.3%)
**4**^**th**^ **Year**	83 (18.1%)	33 (21.4%)	116 (18.9%)
**5**^**th**^ **Year**	57 (12.4%)	-	57 (9.3%)

It can be observed that the samples were mostly composed of female students in both countries, however there was a more balanced gender distribution in the US sample, compared to the Brazilian sample. The mean age of entire sample was 21,5 years old.

The mean values of the USEI items varied between 1.95 (±1.134) and 4.45 (±0.801). The skewness values were considered adequate for all items. Regarding kurtosis, the value of item 3 for the sample of USA students was higher than that recommended (S1 Appendix).

In the Brazilian sample, the items on which the majority of students scored high were 3, 8, 9, and 11, all of them belonging to the behavioral engagement factor. Although students reported having problems with some teachers and classmates (item 8), they reported helping their classmates when they asked them to explain something in their domain (item 11). Additionally, more than half of the students scored 4 on item 1 ("I pay attention in class") (S2 Appendix).

Regarding the USA sample, the items punctuated with higher scores were 3, 4 and 11, all of them belonging to the behavioral engagement factor. Unlike Brazilian students, however, most USA students reported having no problems with professors and classmates.

Table 2 presents the local and global goodness-of-fit indices for the USEI according to the factorial structure of the evaluated samples.

**Table 2:** Confirmatory factor analysis, average variance extracted (AVE), and reliability (composite reliability [CR] and α) of the University Student Engagement Inventory (USEI) administered to dental students.

	Tri-factorial Structure					
	Original		Reduced (15 items)		Reduced refined	
	Brazil	USA	Brazil	USA	Brazil	USA
**Number of items**	32	32	15	15	14	14
**Excluded items**	-	-	-	-	14	5
**Correlation between items**	-	-	-	-	28 e 32 (0.316)	-
**λ**	0.171–0.887	0.258–0.818	0.316–0.906	0.375–0.894	0.499–0.904	0.393–0.897
**Χ** ^**2**^ **/df**	4.38	2.257	2.65	2.351	2.69	2.215
**CFI**	0.802	0.794	0.964	0.872	0.968	0.898
**TLI**	0.786	0.778	0.956	0.846	0.961	0.875
**RMSEA**	0.086	0.090	0.060	0.094	0.061	0.089
**WRMR**	1.995	1.564	1.027	1.095	0.975	1.000
**r**	0.536–0.663	0.454–0.593	0.505–0.576	0.420–0.599	0.532–0.597	0.422–0.571
**AVE**	0.234–0.392	0.325–0.462	0.359–0.525	0.312–0.433	0.359–0.629	0.312–0.430
**CR**	0.749–0.875	0.834–0.892	0.733–0.835	0.680–0.778	0.733–0.870	0.681–0.775
**α**	0.711–0.760	0.623–0.795	0.581–0.666	0.411–0.611	0.479–0.666	0.411–0.611

It can be observed that the 32-item USEI model did not show good global or local fit for the sample of Brazilian dental students. As for the 15-item USEI model proposed by Maroco et al. [1] no good local fit was obtained (item 14: λ = 0.316). When inspecting the LM, a high value was observed between the errors of item 28 and 32 showing similarity between them. So, we inserted a correlation between the errors of items 28 and 32. After inserting this correlation and removing item 14 an adequate fit of the model was obtained for Brazilian sample. The 32-item model presented limited convergent validity, while the other models presented convergent validity with values closer to the recommended. The internal consistency was limited in the 15-item and in the refined model.

For the sample of USA university students, the 32-item and the 15-item USEI models also did not present adequate fit. When removing item 5 (λ = 0.375), the refined 15-item USEI model showed a better adjustment to the sample, despite the limited convergent validity and internal consistency.

Considering the general and local goodness-of-fit values obtained for the analyzed models, it was decided to evaluate the contribution of the independent variables (gender and academic level) to university engagement using only the refined reduced USEI versions (Table 3), since they presented the best fit to each sample.

**Table 3:** Contribution of gender and academic level in the refined reduced USEI factors.

Country	Factor	Variable	β	Standardized β	Standard error	p
**Brazil**	Behavioural Engagement	Gender	0.235	0.170	0.074	0.002
		Academic level	-0.128	-0.281	0.026	<0.001
	Emotional Engagement	Gender	-0.069	-0.063	0.069	0.323
		Academic level	-0.005	-0.014	0.024	0.833
	Cognitive Engagement	Gender	0.057	0.041	0.074	0.443
		Academic level	-0.022	-0.048	0.025	0.388
	Behavioural Engagement	Gender	0.352	0.261	0.134	0.009
		Academic level	0.070	0.115	0.058	0.228
**United States**	Emotional Engagement	Gender	0.039	0.046	0.074	0.603
		Academic level	0.077	0.201	0.036	0.030
	Cognitive Engagement	Gender	0.259	0.273	0.102	0.011
		Academic level	-0.024	-0.055	0.035	0.493

There was a significant effect of gender on behavioral engagement for both samples. For the sample of USA students, there was also a gender influence on cognitive engagement. Academic level was related to behavioral engagement for the Brazilian sample, and to emotional engagement for the USA sample.

**Fig 1:**
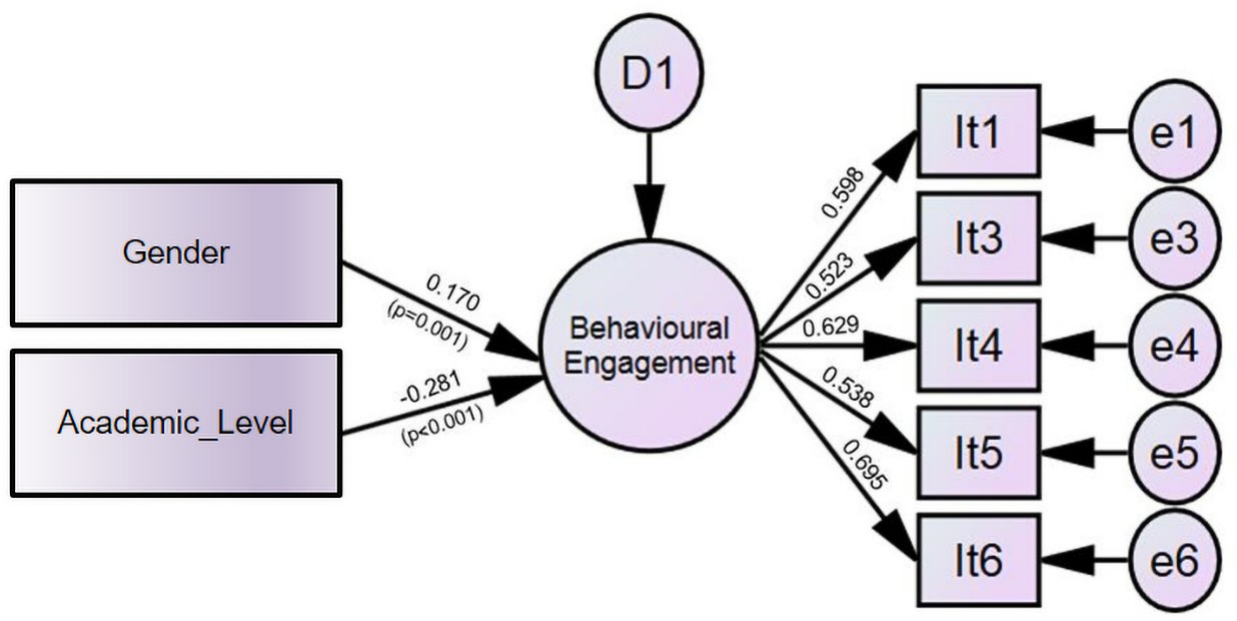
Structural model of variables with a significant contribution (p<0.05) to university engagement in the Brazilian sample.

Fig 1 shows the structural model of the variables with a significant contribution to university engagement in the sample of Brazilian dental students.

In the Brazilian sample, it was observed that gender contributed to behavioral engagement (p = 0.002), with female students presenting greater engagement (standardized β = 0.170). Academic level was related to the same factor (p˂0.001), and the lower the year of the course, the greater the students’ engagement (standardized β = -0.281).

**Fig 2:**
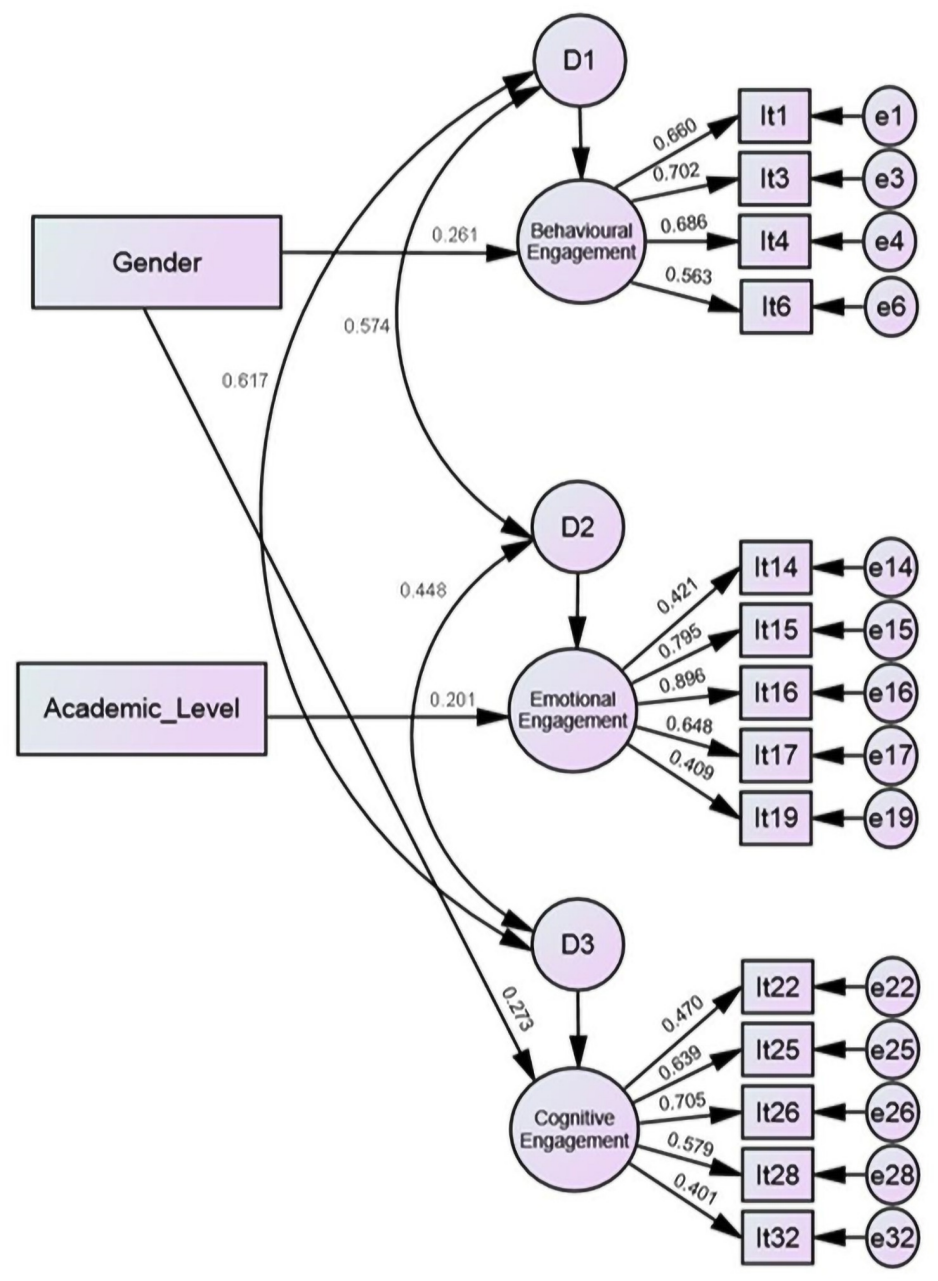
Structural model of variables with a significant contribution (p<0.05) to university engagement in the USA sample.

Fig 2 presents the structural model of the variables with a significant contribution to university engagement in the sample of USA dental students.

Gender was related to behavioral and cognitive engagement (p = 0.009 and 0.011, respectively), with female students presenting greater engagement (standardized β = 0.261 and 0.273, respectively). Academic level was related to emotional engagement (p = 0.030), with students in higher course years presenting greater engagement (standardized β = 0.201).

## Discussion

This study aimed to validate the USEI in its original and reduced tri-factorial structure. The fit was not adequate for the original version in both samples. Subsequently, the psychometric properties of the reduced version were evaluated, which also did not present a good adjustment. For the sample of Brazilian students, after refinement of the reduced version by excluding item 14 (“I don’t feel very accomplished at this school”), which presented low factorial weight (λ = 0.316), and inserting a correlation between the errors of items 28 and 32, the model fit adequately to the sample, despite having limited internal consistency. It is possible that this sample did not clearly understand what it would be like to “feel accomplished”. For the sample of USA students, the refinement of the reduced structure involved removing the item 5 (“When I have doubts, I ask questions and participate in debates in the classroom”), which improved the model fit to the sample. It is possible that there was some misunderstanding in the relationship between clarification of doubts and participation in debates in the classroom. Maroco et al. [1] also did not observe a good sample adjustment for the original 32-item version of USEI. Therefore, they performed a refinement that resulted in the reduced version of the USEI consisting of 15 items. Maroco et al. [1] and Zucoloto et al. [5] found a good fit when using the 15-item USEI with university students and there was no need for further refinement.

In this study, the refined reduced versions of the USEI showed appropriate fit and were therefore chosen for further analysis. In these versions, the item with the highest score for students from both countries was item 3 (“I follow the school’s rules”). This may be related to three factors; one is the perception that individuals have regarding the observance of the proposed rules, which may not be the same as that of their professors and institutions; the other related issue is the profile of health students, who are less politicized than students in the field of humanities and therefore accept the proposed rules without much questioning; the last one may be related to social desirability. According to Pegden and Tucker [16], students’ perceptions may differ depending on the nature of the course and the students’ characteristics. It is seen in this case that the cultural differences between both evaluated countries did not interfere in this aspect.

Regarding the contribution of gender and academic level to the USEI factors, several relationships were verified in both samples. Gender had an influence on the cognitive engagement factor for the USA students, and on the behavioral engagement factor for the samples from both countries, with female students showing greater engagement. Regarding behavioral engagement, no cultural interference was observed, that is, females in both countries showed greater participation in the classroom and extracurricular tasks. On the other hand, females in the USA sample showed greater efforts and willingness to master issues and complex skills. Contreras and Villalobos [17] also verified that females tended to value their education more and spend more time involved in their studies in a sample of Chilean dental students. In a study with Portuguese medical students, Salgueira et al. [18] found that females were more focused on their academic performance, investing more time and effort in issues related to their curricular training. Additionally, Adam et al. [19] observed that English female medical students performed better in many aspects. On the other hand, Borgoyne et al. [20] observed that male Irish medical students had more positive perceptions of skill transfer.

Cultural differences were also evidenced in the relationship between academic level and university engagement. In the USA sample, academic level affected emotional engagement, with higher academic level being associated with higher engagement. Contreras and Villalobos [17] obtained similar results and suggested as a cause the maturation of students throughout the course and the increased contact with the clinical stage of the course, demanding greater responsibility when applying all contents learned to patients. In the Brazilian sample, academic level was related to behavioral engagement, and in this case, the lower the academic level, the greater the engagement. This fact may be related to the curriculum structure in Brazil. In the early years of the course, subjects are general and may require greater involvement of the students with classroom tasks. Once the subjects are not specifically related to the dental practice, students can search for extracurricular activities to keep themselves motivated. In addition, course requirements over time, patient care, and internships could cause Brazilian students to have less engagement with their academic activities due to overload.

According to Zucoloto et al. [5] and Maroco et al. [1] school engagement is an antidote for low performance, protecting students from occupational and psychosocial risks. Capri et al. [21] emphasized that school engagement contributes to the healthy development of students and consequently to their academic success. Thus, educational institutions should direct their efforts to provide support strategies and encouragement for students to remain involved with academic activities throughout the course [6].

In this study, the best-fitting USEI structure and the influence of gender and academic level on the evaluated construct differed between USA and Brazilian students, which may be related to socio-cultural differences. Schaufeli et al. [4] emphasized the importance of conducting cross-national studies to compare psychometric properties of instruments between samples from different countries. Furthermore, as the USEI was developed and initially tested in a sample of Portuguese university students, it needs to be used in transnational studies to verify its psychometric structure before it is used in interventions to promote student engagement, ultimately aiming to improve performance, reduce academic failure, and promote psychological well-being [1].

This study has some limitations. The first one is related to the cross-sectional study design, which is non-analytical observational and prevents the inference of causality between variables. Another limitation was the small sample of USA students and the use of convenience sampling, which prevents the generalization of results. Despite these limitations, this is the first study to evaluate university engagement using the USEI with dental students; therefore, the results contribute to the field of dental education. Another important contribution is the transnational nature of this research, showing that caution should be exercised when using the USEI with different nationalities.

Further studies should be conducted using the USEI with an analytical observational approach, using a probabilistic sample, including dental students of other countries and using measuring instruments to simultaneously observe stress, depression and burnout syndrome.

## Conclusion

The refined reduced version of the USEI presented better psychometric properties than did the original and other reduced versions. Gender influenced cognitive engagement in the USA sample and behavioral engagement in both samples, with female students presenting greater engagement. Academic level influenced behavioral engagement in the Brazilian sample, with students in the first years of the course showing greater involvement, and on emotional engagement in the USA sample, with students in the last years of the course demonstrating greater involvement.

## Supporting information

S1 Appendix Psychometric sensitivity of USEI items. (DOCX)

S2 Appendix Distribution of student answers to the USEI items. (DOCX)
