# Correction: University engagement of dental students related to educational environment: A transnational study

**DOI:** 10.1371/journal.pone.0305676

**Published:** 2024-06-13

**Authors:** Cristina Dupim Presoto, Danielle Wajngarten, Patrícia Aleixo dos Santos Domingos, Ana Carolina Botta, Juliana Alvares Duarte Bonini Campos, Júlia Margato Pazos, Patrícia Petromilli Nordi Sasso Garcia

S1 Appendix did not include attribution to the original source as required by the CC-BY 4.0 license. Please view the updated S1 Appendix below.

There is an error in reference 1. The correct reference is: Maroco J, Maroco AL, Campos JADB, Fredricks JA. University student’s engagement: development of the University Student Engagement Inventory (USEI). Psicol. Refl. Crít. 2016; 29(21):1–12.

## Supporting information

S1 Appendix Psychometric sensitivity of USEI items. (DOCX)
