# Supplementary material for: University engagement of dental students related to educational environment: A transnational study
Source: PLoS One. 2021 Nov 4;16(11):e0259524. doi: 10.1371/journal.pone.0259524 (PMC8568266; doi:10.1371/journal.pone.0259524)
Supplement: S2 Appendix — (DOCX) [file pone.0259524.s002.docx]

**Appendix 2.** Distribution of student answers to the USEI items.

|  | **n (%)** | | | | | | | | | | | | | | | | | | | |
| --- | --- | --- | --- | --- | --- | --- | --- | --- | --- | --- | --- | --- | --- | --- | --- | --- | --- | --- | --- | --- |
| **Items** | **1** | | | | **2** | | |  | **3** | | | | **4** | | | | **5** | | | |
|  | **Brazil** | | **USA** | | **Brazil** | | **USA** | | **Brazil** | | **USA** | | **Brazil** | | **USA** | | **Brazil** | | **EUA** | |
| BE1 | 4 | 0.9 | 4 | 2.6 | 28 | 6.1 | 14 | 9.1 | 115 | 25.1 | 51 | 33.1 | 248 | 54.0 | 71 | 46.1 | 64 | 13.9 | 14 | 9.1 |
| BE2 | 30 | 6.5 | 13 | 8.4 | 85 | 18.5 | 30 | 19.5 | 121 | 26.4 | 38 | 24.7 | 141 | 30.7 | 50 | 32.5 | 82 | 17.9 | 23 | 14.9 |
| BE3 | 5 | 1.1 | 2 | 1.3 | 15 | 3.3 | 3 | 1.9 | 48 | 10.5 | 9 | 5.8 | 149 | 32.5 | 49 | 31.8 | 242 | 52.7 | 91 | 59.1 |
| BE4 | 11 | 2.4 | 1 | 0.6 | 58 | 12.6 | 4 | 2.6 | 84 | 18.3 | 16 | 10.4 | 162 | 35.3 | 50 | 32.5 | 144 | 31.4 | 83 | 53.9 |
| BE5 | 64 | 13.9 | 17 | 11.0 | 145 | 31.6 | 20 | 13.0 | 129 | 28.1 | 57 | 37.0 | 80 | 17.4 | 37 | 24.0 | 41 | 8.9 | 23 | 14.9 |
| BE6 | 9 | 2.0 | 3 | 1.9 | 39 | 8.5 | 9 | 5.8 | 65 | 14.2 | 35 | 22.7 | 164 | 35.7 | 56 | 36.4 | 182 | 39.7 | 51 | 33.1 |
| BE7R | 52 | 11.3 | 10 | 6.5 | 93 | 20.3 | 41 | 26.6 | 154 | 33.6 | 49 | 31.8 | 112 | 24.4 | 42 | 27.3 | 48 | 10.5 | 12 | 7.8 |
| BE8R | 10 | 2.2 | 55 | 35.7 | 13 | 2.8 | 58 | 37.7 | 53 | 11.5 | 23 | 14.9 | 119 | 25.9 | 8 | 5.2 | 264 | 57.5 | 10 | 6.5 |
| BE9R | 12 | 2.6 | 80 | 51.9 | 14 | 3.1 | 44 | 28.6 | 44 | 9.6 | 24 | 15.6 | 141 | 30.7 | 6 | 3.9 | 248 | 54.0 | - | - |
| BE10 | 8 | 1.7 | 5 | 3.2 | 45 | 9.8 | 12 | 7.8 | 116 | 25.3 | 48 | 31.2 | 139 | 30.3 | 54 | 35.1 | 151 | 32.9 | 35 | 22.7 |
| BE11 | 5 | 1.1 | - | - | 13 | 2.8 | 3 | 1.9 | 61 | 13.3 | 20 | 13.0 | 111 | 24.2 | 53 | 34.4 | 269 | 58.6 | 78 | 50.6 |
| EE12 | 39 | 8.5 | 13 | 8.4 | 90 | 19.6 | 27 | 17.5 | 185 | 40.3 | 42 | 27.3 | 95 | 20.7 | 34 | 22.1 | 50 | 10.9 | 38 | 24.7 |
| EE13 | 6 | 1.3 | 7 | 4.5 | 27 | 5.9 | 19 | 12.3 | 68 | 14.8 | 44 | 28.6 | 208 | 45.3 | 64 | 41.6 | 150 | 32.7 | 20 | 13.0 |
| EE14R | 29 | 6.3 | 46 | 29.9 | 44 | 9.6 | 50 | 32.5 | 54 | 11.8 | 37 | 24.0 | 132 | 28.8 | 14 | 9.1 | 200 | 43.6 | 7 | 4.5 |
| EE15 | 16 | 3.5 | 19 | 12.3 | 56 | 12.2 | 39 | 25.3 | 112 | 24.4 | 59 | 38.3 | 162 | 35.3 | 36 | 23.4 | 113 | 24.6 | 1 | 0.6 |
| EE16 | 12 | 2.6 | 8 | 5.2 | 29 | 6.3 | 27 | 17.5 | 96 | 20.9 | 65 | 42.2 | 174 | 37.9 | 39 | 25.3 | 148 | 32.2 | 15 | 9.7 |
| EE17 | 9 | 2.0 | 6 | 3.9 | 36 | 7.8 | 18 | 11.7 | 82 | 17.9 | 46 | 29.9 | 175 | 38.1 | 64 | 41.6 | 157 | 34.2 | 20 | 13.0 |
| EE18 | 88 | 19.2 | 28 | 18.2 | 127 | 27.7 | 38 | 24.7 | 111 | 24.2 | 37 | 24.0 | 71 | 15.5 | 38 | 24.7 | 62 | 13.5 | 13 | 8.4 |
| EE19 | 28 | 6.1 | 12 | 7.8 | 90 | 19.6 | 34 | 22.1 | 150 | 32.7 | 71 | 46.1 | 144 | 31.4 | 30 | 19.5 | 47 | 10.2 | 7 | 4.5 |
| EE20 | 139 | 30.3 | 22 | 14.3 | 124 | 27.0 | 20 | 13.0 | 93 | 20.3 | 48 | 31.2 | 49 | 10.7 | 30 | 19.5 | 54 | 11.8 | 34 | 22.1 |
| EE21 | 51 | 11.1 | 11 | 7.1 | 120 | 26.1 | 22 | 14.3 | 144 | 31.4 | 48 | 31.2 | 85 | 18.5 | 55 | 35.7 | 59 | 12.9 | 18 | 11.7 |
| CE22 | 24 | 5.2 | 9 | 5.8 | 71 | 15.5 | 34 | 22.1 | 128 | 27.9 | 55 | 35.7 | 134 | 29.2 | 41 | 26.6 | 102 | 22.2 | 15 | 9.7 |
| CE23 | 98 | 21.4 | 27 | 17.5 | 150 | 32.7 | 42 | 27.3 | 125 | 27.2 | 45 | 29.2 | 54 | 11.8 | 25 | 16.2 | 32 | 7.0 | 15 | 9.7 |
| CE24 | 119 | 25.9 | 71 | 46.1 | 142 | 30.9 | 42 | 27.3 | 114 | 24.8 | 26 | 16.9 | 59 | 12.9 | 7 | 4.5 | 25 | 5.4 | 8 | 5.2 |
| CE25 | 17 | 3.7 | 19 | 12.3 | 58 | 12.6 | 26 | 16.9 | 148 | 32.2 | 51 | 33.1 | 141 | 30.7 | 41 | 26.6 | 95 | 20.7 | 17 | 11.0 |
| CE26 | 16 | 3.5 | 5 | 3.2 | 42 | 9.2 | 8 | 5.2 | 73 | 15.9 | 31 | 20.1 | 151 | 32.9 | 65 | 42.2 | 177 | 38.6 | 45 | 29.2 |
| CE27 | 28 | 6.1 | 5 | 3.2 | 52 | 11.3 | 12 | 7.8 | 116 | 25.3 | 49 | 31.8 | 117 | 25.5 | 54 | 35.1 | 146 | 31.8 | 34 | 22.1 |
| CE28 | 10 | 2.2 | 6 | 3.9 | 30 | 6.5 | 15 | 9.7 | 135 | 29.4 | 50 | 32.5 | 171 | 37.3 | 58 | 37.7 | 113 | 24.6 | 25 | 16.2 |
| CE29 | 42 | 9.2 | 27 | 17.5 | 126 | 27.5 | 47 | 30.5 | 163 | 35.5 | 47 | 30.5 | 93 | 20.3 | 25 | 16.2 | 35 | 7.6 | 8 | 5.2 |
| CE30 | 4 | 0.9 | 1 | 0.6 | 29 | 6.3 | 13 | 8.4 | 69 | 15.0 | 33 | 21.4 | 146 | 31.8 | 65 | 42.2 | 211 | 46.0 | 42 | 27.3 |
| CE31 | 51 | 11.1 | 12 | 7.8 | 125 | 27.2 | 34 | 22.1 | 145 | 31.6 | 35 | 22.7 | 93 | 20.3 | 46 | 29.9 | 45 | 9.8 | 27 | 17.5 |
| CE32 | 10 | 2.2 | 7 | 4.5 | 43 | 9.4 | 18 | 11.7 | 133 | 29.0 | 42 | 27.3 | 160 | 34.9 | 61 | 39.6 | 113 | 24.6 | 26 | 16.9 |
