# Supplementary material for: Correction: University engagement of dental students related to educational environment: A transnational study
Source: PLoS One. 2024 Jun 13;19(6):e0305676. doi: 10.1371/journal.pone.0305676 (PMC11175497; doi:10.1371/journal.pone.0305676)
Supplement: S1 Appendix — (DOCX) [file pone.0305676.s001.docx]

**Appendix 1.** Psychometric sensitivity of USEI items.

| **Items** | | **Samples** | | | | | | | | | | | | | | |
| --- | --- | --- | --- | --- | --- | --- | --- | --- | --- | --- | --- | --- | --- | --- | --- | --- |
|  |  | **Mean** | | |  | **Standard deviation** | | |  | **Skewness** | | |  | **Kurtosis** | | |
|  |  | **Brazil** | **USA** | **Total** |  | **Brazil** | **USA** | **Total** |  | **Brazil** | **USA** | **Total** |  | **Brazil** | **USA** | **Total** |
| BE1 | I pay attention in class. | 3.74 | 3.50 | 3.68 |  | 0.804 | 0.880 | 0.830 |  | -0.610 | -0.583 | -0.619 |  | 0.575 | 0.403 | 0.547 |
|  | (Eu estou atento na aula). |  |  |  |  |  |  |  |  |  |  |  |  |  |  |  |
| BE2 | When I’m in class, I behave as if it was a job. | 3.35 | 3.26 | 3.33 |  | 1.162 | 1.182 | 1.166 |  | -0.271 | -0.278 | -0.273 |  | -0.797 | -0.826 | -0.804 |
|  | (Quando estou em sala de aula, eu me comporto como se estivesse num emprego). |  |  |  |  |  |  |  |  |  |  |  |  |  |  |  |
| BE3 | I follow the school’s rules. | 4.32 | 4.45 | 4.36 |  | 0.870 | 0.801 | 0.854 |  | -1.380 | -1.859 | -1.482 |  | 1.803 | 4.272 | 2.245 |
|  | (Eu sigo as regras da escola). |  |  |  |  |  |  |  |  |  |  |  |  |  |  |  |
| BE4 | I usually do my homework on time. | 3.81 | 4.36 | 3.95 |  | 1.088 | 0.823 | 1.055 |  | -0.652 | -1.336 | -0.814 |  | -0.447 | 1.721 | -0.147 |
|  | (Geralmente faço os trabalhos de casa em dia (a tempo e horas)). |  |  |  |  |  |  |  |  |  |  |  |  |  |  |  |
| BE5 | When I have doubts I ask questions and participate  in debates in the classroom. | 2.76 | 3.19 | 2.87 |  | 1.162 | 1.176 | 1.179 |  | 0.288 | -0.226 | 0.159 |  | -0.730 | -0.593 | -0.800 |
|  | (Quando tenho dúvidas, faço perguntas e envolvo-me nos debates da sala de aula). |  |  |  |  |  |  |  |  |  |  |  |  |  |  |  |
| BE6 | I usually participate actively in group assignments. | 4.03 | 3.93 | 4.00 |  | 1.028 | 0.984 | 1.017 |  | -0.962 | -0.731 | -0.901 |  | 0.249 | 0.116 | 0.188 |
|  | (Geralmente participo ativamente nos trabalhos de grupo). |  |  |  |  |  |  |  |  |  |  |  |  |  |  |  |
| BE7R | I usually go to class without having read the materials  recommended by Professor. | 3.02 | 2.97 | 3.01 |  | 1.149 | 1.057 | 1.126 |  | -0.082 | -0.002 | -0.061 |  | -0.725 | -0.694 | -0.714 |
|  | (Geralmente vou para as aulas sem ter lido os materiais recomendados pelo professor). |  |  |  |  |  |  |  |  |  |  |  |  |  |  |  |
| BE8R | I have problems with some teachers at school. | 4.34 | 3.91 | 4.23 |  | 0.942 | 1.140 | 1.012 |  | -1.553 | -1.108 | -1.432 |  | 2.175 | 0.631 | 1.681 |
|  | (Eu tenho problemas com alguns professores na escola). |  |  |  |  |  |  |  |  |  |  |  |  |  |  |  |
| BE9R | I have problems with other colleagues. | 4.31 | 4.29 | 4.30 |  | 0.950 | 0.869 | 0.930 |  | -1.594 | -0.955 | -1.463 |  | 2.451 | -0.068 | 1.976 |
|  | (Eu tenho problemas com outros colegas da escola). |  |  |  |  |  |  |  |  |  |  |  |  |  |  |  |
| BE10 | I ask for help from colleagues when I do not understand any of the materials of classes. | 3.83 | 3.66 | 3.79 |  | 1.050 | 1.018 | 1.044 |  | -0.525 | -0.487 | -0.506 |  | -0.561 | -0.113 | -0.472 |
|  | (Peço ajuda a colegas quando não entendo/percebo alguma das matérias das aulas). |  |  |  |  |  |  |  |  |  |  |  |  |  |  |  |
| BE11 | I help colleagues when they ask me to explain subjects I understand well. | 4.36 | 4.34 | 4.36 |  | 0.894 | 0.777 | 0.866 |  | -1.389 | -0.925 | -1.305 |  | 1.489 | 0.093 | 1.290 |
|  | (Eu ajudo os colegas quando me pedem para explicar algo que eu entendo/percebo bem). |  |  |  |  |  |  |  |  |  |  |  |  |  |  |  |
| EE12 | I attend extracurricular activities in my school (concerts, exhibitions, lectures, conferences …). | 3.06 | 3.37 | 3.14 |  | 1.085 | 1.263 | 1.139 |  | -0.004 | -0.236 | -0.031 |  | -0.471 | -0.974 | -0.653 |
|  | (Eu assisto às atividades extracurriculares da minha escola (concertos, peças, exposições, palestras, conferências etc)). |  |  |  |  |  |  |  |  |  |  |  |  |  |  |  |
| EE13 | I am happy at this school. | 4.02 | 3.46 | 3.88 |  | 0.911 | 1.017 | 0.969 |  | -0.932 | -0.535 | -0.820 |  | 0.722 | -0.119 | 0.368 |
|  | (Sinto-me feliz nesta escola). |  |  |  |  |  |  |  |  |  |  |  |  |  |  |  |
| EE14R | I don’t feel very accomplished at this school. | 3.94 | 3.74 | 3.89 |  | 1.227 | 1.119 | 1.203 |  | -1.020 | -0.661 | -0.925 |  | -0.017 | -0.241 | -0.119 |
|  | (Sinto-me pouco realizado nesta escola). |  |  |  |  |  |  |  |  |  |  |  |  |  |  |  |
| EE15 | I feel excited about the school work. | 3.65 | 2.75 | 3.43 |  | 1.084 | 0.974 | 1.127 |  | -0.518 | -0.245 | -0.350 |  | -0.444 | -0.764 | -0.633 |
|  | (Sinto-me entusiasmado com o trabalho da escola). |  |  |  |  |  |  |  |  |  |  |  |  |  |  |  |
| EE16 | I like being at school. | 3.91 | 3.17 | 3.72 |  | 1.007 | 1.002 | 1.055 |  | -0.809 | -0.69 | -0.568 |  | 0.252 | -0.263 | -0.253 |
|  | (Eu gosto de estar na escola). |  |  |  |  |  |  |  |  |  |  |  |  |  |  |  |
| EE17 | I am interested in the school work. | 3.95 | 3.48 | 3.83 |  | 1.005 | 0.992 | 1.021 |  | -0.828 | -0.516 | -0.708 |  | 0.136 | -0.055 | -0.041 |
|  | (Estou interessado no trabalho da escola). |  |  |  |  |  |  |  |  |  |  |  |  |  |  |  |
| EE18 | I usually talk to teachers about my professional interests/career. | 2.76 | 2.81 | 2.77 |  | 1.299 | 1.237 | 1.283 |  | 0.288 | 0.063 | 0.235 |  | -0.990 | -1.050 | -1.006 |
|  | (Eu costumo falar com professores sobre os meus interesses profissionais/carreira). |  |  |  |  |  |  |  |  |  |  |  |  |  |  |  |
| EE19 | My classroom is an interesting place to be. | 3.20 | 2.91 | 3.13 |  | 1.061 | 0.952 | 1.042 |  | -0.198 | -0.046 | -0.135 |  | -0.584 | -0.092 | -0.514 |
|  | (Minha sala de aula é um lugar interessante para estar). |  |  |  |  |  |  |  |  |  |  |  |  |  |  |  |
| EE20 | I get involved in extracurricular activities with other  members of the school community outside of the classroom (cultural groups, student associations, sports groups,...) | 2.47 | 3.22 | 2.66 |  | 1.333 | 1.320 | 1.369 |  | 0.572 | -0.208 | 0.358 |  | -0.813 | -0.973 | -1.061 |
|  | (Envolvo-me em atividades extracurriculares com outros membros da comunidade escolar fora do âmbito das aulas (grupos culturais, associações estudantis, grupos desportivos)). |  |  |  |  |  |  |  |  |  |  |  |  |  |  |  |
| EE21 | I discuss with my colleagues about possible ways to  improve our coursework/school. | 2.96 | 3.31 | 3.05 |  | 1.186 | 1.081 | 1.169 |  | 0.143 | -0.414 | 0.003 |  | -0.810 | -0.387 | -0.812 |
|  | (Discuto com os meus colegas sobre possíveis formas de melhorar o nosso curso/escola). |  |  |  |  |  |  |  |  |  |  |  |  |  |  |  |
| CE22 | When I read a book, I question myself to make sure I understand the subject I’m reading about. | 3.48 | 3.12 | 3.39 |  | 1.149 | 1.050 | 1.134 |  | -0.330 | -0.044 | -0.237 |  | -0.725 | -0.557 | -0.741 |
|  | (Quando leio um livro, questiono-me para ter certeza que entendo o assunto que estou a ler). |  |  |  |  |  |  |  |  |  |  |  |  |  |  |  |
| CE23 | I study at home even when I do not have assessment  tests. | 2.50 | 2.73 | 2.56 |  | 1.155 | 1.210 | 1.169 |  | 0.488 | 0.257 | 0.429 |  | -0.492 | -0.790 | -0.590 |
|  | (Eu estudo em casa mesmo quando não tenho testes de avaliação). |  |  |  |  |  |  |  |  |  |  |  |  |  |  |  |
| CE24 | I try to watch TV programs on subjects that we are talking about in class. | 2.41 | 1.95 | 2.30 |  | 1.161 | 1.134 | 1.170 |  | 0.494 | 1.153 | 0.626 |  | -0.593 | 0.645 | -0.465 |
|  | (Eu tento assistir a programas de TV sobre matérias que estamos a aprender nas aulas). |  |  |  |  |  |  |  |  |  |  |  |  |  |  |  |
| CE25 | I talk to people outside the school on matters that I learned in class. | 3.52 | 3.07 | 3.41 |  | 1.068 | 1.172 | 1.111 |  | -0.313 | -0.190 | -0.311 |  | -0.522 | -0.720 | -0.541 |
|  | (Eu converso com outras pessoas fora da escola sobre as matérias que aprendo nas aulas). |  |  |  |  |  |  |  |  |  |  |  |  |  |  |  |
| CE26 | If I do not understand the meaning of a word, I try to solve the problem, for example by consulting a dictionary or asking someone else. | 3.94 | 3.89 | 3.93 |  | 1.106 | 0.994 | 1.078 |  | -0.910 | -0.909 | -0.905 |  | 0.038 | 0.692 | 0.159 |
|  | (Se não compreendo o significado de uma palavra, eu tento resolver o problema, por exemplo, consultando um dicionário ou perguntando a outra pessoa). |  |  |  |  |  |  |  |  |  |  |  |  |  |  |  |
| CE27 | I check my homework to correct for errors. | 3.66 | 3.65 | 3.65 |  | 1.207 | 1.013 | 1.161 |  | -0.541 | -0.472 | -0.531 |  | -0.642 | -0.107 | -0.525 |
|  | (Eu verifico os meus trabalhos escolares para corrigir erros). |  |  |  |  |  |  |  |  |  |  |  |  |  |  |  |
| CE28 | I try to integrate the acquired knowledge in solving new problems. | 3.76 | 3.53 | 3.70 |  | 0.970 | 1.005 | 0.983 |  | -0.505 | -0.464 | -0.495 |  | -0.074 | -0.053 | -0.071 |
|  | (Tento integrar os conhecimentos adquiridos para resolver problemas novos). |  |  |  |  |  |  |  |  |  |  |  |  |  |  |  |
| CE29 | I read other books or materials to learn more about the subjects we discuss in class. | 2.90 | 2.61 | 2.83 |  | 1.068 | 1.110 | 1.085 |  | 0.130 | 0.269 | 0.151 |  | -0.556 | -0.630 | -0.594 |
|  | (Eu leio outros livros ou materiais para aprender mais sobre as matérias que temos nas aulas). |  |  |  |  |  |  |  |  |  |  |  |  |  |  |  |
| CE30 | If I do not understand something I read, I go back and  read it again. | 4.16 | 3.87 | 4.08 |  | 0.958 | 0.934 | 0.960 |  | -0.991 | -0.567 | -0.860 |  | 0.271 | -0.250 | 0.030 |
|  | (Se eu não entender/perceber algo que li, volto atrás e leio de novo). |  |  |  |  |  |  |  |  |  |  |  |  |  |  |  |
| CE31 | I review my notes/materials after the school classes. | 2.90 | 3.27 | 3.00 |  | 1.142 | 1.211 | 1.170 |  | 0.136 | -0.204 | 0.062 |  | -0.739 | -0.968 | -0.851 |
|  | (Revejo os meus apontamentos/materiais depois das aulas). |  |  |  |  |  |  |  |  |  |  |  |  |  |  |  |
| CE32 | I try to integrate subjects from different disciplines into my general knowledge. | 3.70 | 3.53 | 3.66 |  | 1.011 | 1.049 | 1.022 |  | -0.438 | -0.534 | -0.467 |  | -0.369 | -0.189 | -0.300 |
|  | (Tento integrar as matérias das diferentes disciplinas no meu conhecimento geral). |  |  |  |  |  |  |  |  |  |  |  |  |  |  |  |

This file includes the University Student Engagement Inventory (USEI), originally developed by Maroco J, Maroco AL, Campos JADB, Fredricks JA (2016). University student’s engagement: development of the University Student Engagement Inventory (USEI). Psicol Refl Crít 29(21): 1-12. <https://doi.org/10.1186/s41155-016-0042-8>
